# *Xenomyrothecium tongaense* PTS8: a rare endophyte of *Polianthes tuberosa* with salient antagonism against multidrug-resistant pathogens

**DOI:** 10.3389/fmicb.2024.1327190

**Published:** 2024-02-16

**Authors:** Ranjitha Dhevi V. Sundar, Sathiavelu Arunachalam

**Affiliations:** ^1^Laboratory of Microbiology, Department of Biotechnology, School of Biosciences and Technology, Vellore Institute of Technology, Vellore, India; ^2^Laboratory of Microbiology, Department of Agriculture Microbiology, VIT School of Agricultural Innovations and Advanced Learning, Vellore Institute of Technology, Vellore, India

**Keywords:** pharmacology, fungal metabolites, *Xenomyrothecium tongaense*, antibiotic resistance, multi-drug-resistant, antibacterial agent, agar plug diffusion assay

## Abstract

**Introduction:**

Endophytes refer to microorganisms residing within the endosphere of plants, particularly perennials, without inflicting noticeable injury or inducing obvious morphological variations to their host plant or host organism. Endophytic fungi, although often overlooked microorganisms, have garnered interest due to their significant biological diversity and ability to produce novel pharmacological substances.

**Methods:**

In this study, fourteen endophytic fungi retrieved were from the stem of the perennial plant *Polianthes tuberosa* of the Asparagaceae family. These fungal crude metabolites were tested for antagonistic susceptibility to Multi-Drug Resistant (MDR) pathogens using agar well diffusion, Minimum Inhibitory Concentration (MIC), and Minimum Bactericidal Concentration (MBC) assays. The chequerboard test was used to assess the synergistic impact of active extract.

**Results and discussion:**

In early antibacterial screening using the Agar plug diffusion test, three of fourteen endophytes demonstrated antagonism against Methicillin-resistant *Staphylococcus aureus* (MRSA) and Vancomycin-resistant *Enterococcus* (VRE). Three isolates were grown in liquid medium and their secondary metabolites were recovered using various organic solvents. Eight extracts from three endophytic fungi displayed antagonism against one or more human pathogens with diameters ranging from 11 to 24 mm. The highest antagonistic effect was obtained in ethyl acetate extract for PTS8 isolate against two MRSA (ATCC 43300, 700699) with 20 ± 0.27 and 22 ± 0.47 mm zones of inhibition, respectively, among different solvent extracts. The extract had MICs of 3.12 ± 0.05 and 1.56 ± 0.05 μg/mL, and MBCs of 50 ± 0.01 and 12.5 ± 0.04 μg/mL, respectively. Antagonism against VRE was 18 ± 0.23 mm Zone of Inhibition (ZOI) with MIC and MBC of 6.25 ± 0.25 and 25 ± 0.01 μg/mL. When ethyl acetate extract was coupled with antibiotics, the chequerboard assay demonstrated a synergistic impact against MDR bacteria. In an antioxidant test, it had an inhibitory impact of 87 ± 0.5% and 88.5 ± 0.5% in 2,2-Diphenyl-1-Picrylhydrazyl and reducing power assay, respectively, at 150 μg/mL concentration. PTS8 was identified as a *Xenomyrothecium tongaense* strain by 18S rRNA internal transcribed spacer (ITS) sequencing. To our insight, it is the foremost study to demonstrate the presence of an *X. tongaense* endophyte in the stem of P. tuberosa and the first report to study the antibacterial efficacy of *X. tongaense* which might serve as a powerful antibacterial source against antibiotic-resistant human infections.

## 1 Background

Antimicrobial resistance is not a new phenomenon, but the number of resistant organisms, the geographic areas where drug resistance occurs, and the range of resistance in individual organisms are all unprecedented and escalating. Tuberculosis has been reemerging since the 1980s, and it is now notably multidrug-resistant (MDR), with infection with the human immunodeficiency virus (HIV) exacerbating the disease. Due to the severity of the challenges in treating Multi-Drug Resistant (MDR) strains, the use of many drugs, typically six to seven different medications, is necessary (Levy and Marshall, 2004). However, due to overuse, many antibiotics have lost most of their potency, resulting in more disease-causing bacteria acquiring resistance to them (Abdulmyanova et al., 2020). Antibiotic-producing bacteria have a variety of complicated defense systems to protect themselves from the antibiotics they produce (Peterson and Kaur, 2018). *Staphylococcus aureus*, the dangerous hospital bacterium MRSA (Methicillin-resistant *Staphylococcus aureus*), or ESBL (Extended-spectrum lactamases) bacteria that manufacture beta-lactamases, for example, are resistant to a variety of treatment drugs (Wen et al., 2023). Infectious disorders caused by antibiotic-resistant bacteria are becoming more common, posing a substantial medical problem. Poor aseptic settings, inappropriate treatment usage, late analysis of illnesses, and continual movement of travelers all contributed to the increase in instances (Fuego et al., 2021). *S.aureus*, which is resistant to methicillin, is one of the leading causes of hospital-acquired disease in many Indian hospitals. According to antibiotic susceptibility testing, around 30–80% of them have been documented from various places (Gowrishankar et al., 2013). Over the previous two decades, there has been a rise in the number of MRSA, *Streptococcus pneumoniae* resistant to penicillin, and *Enterococcus faecium* resistant to Vancomycin medicines. Furthermore, recent medicines such as Daptomycin and Linezolid have developed resistance (Deshmukh et al., 2015). As a result, there is an urgent need to prevent antimicrobial resistance by improving antibiotic practice and reducing cross-infection in hospitals. Nonetheless, new antibiotic development must continue since it is critical for sustaining antimicrobial therapy effectiveness (Janeš et al., 2007).

Bioactive chemicals derived from natural sources were regarded as the foundation for high-value product development. Their biological functionality has allowed them to continue to be used in agriculture, medicine, and the food sector (El-Sayed et al., 2022). They were deemed possible sources of active metabolites with a variety of distinct restorative properties, such as steroids, xanthones, phenol, flavonoids, tetralones, alkaloids, benzopyranones, and terpenoids (Soni et al., 2021). Several compounds now in clinical trials were modifications of existing antibiotic classes discovered in recent drug discovery and development programs. They represent only transitory remedies to the growing opposition. As a result, there is a growing interest in identifying chemicals from novel families with distinct structures and action mechanisms to treat drug-resistant infections (Sierra-Zapata et al., 2020). Medicinal plants include a varied range of cultural endophytes capable of producing structurally intriguing and bioactive secondary metabolites (An et al., 2020). Endophytes are microorganisms that live in the plant’s endosphere but do not cause disease symptoms (Mishra and Priyanka, 2022). They colonize the plant tissue, either inter or intracellularly, and maintain a harmonic symbiotic relationship in all of the plants studied (Soni et al., 2021). The host and endophyte interact mutually, with the host providing nutrients and shelter and the endophytes acting as chemical sentries (Ababutain et al., 2021). Interestingly, fungal endophytes and their host plants can produce the same or similar bioactive compounds. As a result, they may be employed to produce a replaceable method for producing beneficial bioactive compounds to safeguard plants and the natural environment (Ye et al., 2021). Fungal endophytes have grabbed the interest of researchers because they provide new sources of antibacterial components, cytotoxic substances such as anti-carcinogenic agents, and bio-stimulants for essential oil production (Alam et al., 2021). Many plant species have been examined because of their diverse endophyte variety and potential to induce bioactive secondary metabolites (Munshi et al., 2021). To yet, practically all higher plants investigated have been related to endophytes that live above and below ground (Mishra and Priyanka, 2022). Over 300,000 plant species on the planet that are developing in new zones host one or more endophytes (Sharma et al., 2016).

*Polianthes tuberosa* L., often referred to as Rajanigandha a plant used for ornamental purposes in the Asparagaceae family. It originated in Mexico and is now grown in a variety of tropical and subtropical climates. Because of aromatic compounds, they are mostly used in the perfume industry. Furthermore, the plant comprises a variety of secondary metabolites, including steroid glycosides and flavonoids, whereas the flower mostly contains terpenoid derivatives and benzoid (Fan et al., 2018; Safeena et al., 2019; Alghuthaymi et al., 2021). As a result, there is an urgent need for novel antibacterial drugs to combat the increasing range of illnesses. The main aim of this study was to evaluate the hostile actions of endophytic fungus from the *P. tuberosa* plant on multi-drug-resistant bacterial infections. The present study goal will lead to a better understanding of endophytic fungi and their use in antibiotic-resistant disease treatment.

## 2 Materials and methodology

### 2.1 Procurement of chemicals and glassware

Chemicals utilized in the experiment were procured from HiMedia Ltd. Mumbai, India. The glassware was from Borosil and the solvents used were acquired from SRL.

### 2.2 Sampling of plant materials for the study of endophytic organisms

To isolate endophytic fungi, a healthy stem of *Polianthes tuberosa* L. has been collected in sterile polyethylene bags from the Vellore district (12°56′44.8”N 79°13′58.2″E), Tamil Nadu, India. Within 12 h after collection, the samples were processed. The plant was certified by Professor Dr. Jayaraman P, PARC (Plant Anatomy Research Centre), West Tambaram, Chennai-600045 (Basha et al., 2012).

### 2.3 Surface sterilization and endophytic fungi isolation

The stem materials were carefully removed from the healthy *P. tuberosa* and washed for 5 min with distilled (d.H_2_O) water to eliminate extraneous debris. The exterior component was then sterilized by serially immersing it in 70% ethanol (C_2_H_6_O) for a minute, 4% sodium hypochlorite (NaOCl) for 5 min, and Milli-Q for a minute. To test the surface sterilization effectiveness, the final washed Milli-Q water was dispersed over the Potato Dextrose Agar (MH096) medium. The stem was then split into small parts 0.5 cm^2^/1 cm long. The explants were transferred and impregnated onto the PDA dish and incubated at 27°C with a daily monitor for 7–10 days till the fungus appeared. Using the single hyphal tip approach, the developing fungus was transferred to a fresh medium. The pure strain was evaluated based on colony morphology and stored in slants at 4°C (Liu et al., 2019).

### 2.4 Endophytic fungal growth rate assessment

The endophytic fungal isolate PTS8 was cultured on a PDA to probe the growth rate of mycelia. The inoculated fungal plate was incubated at 27 ± 2°C for a week. The mycelial growth rate was documented every 24 h by assessing the diameter of the growing mycelium (Techaoei et al., 2020).

### 2.5 The hostile activity of the endophytic fungi

#### 2.5.1 MDR bacterial culture standardization

Pathogenic bacterial strains used were Methicillin-resistant strains of *Staphylococcus aureus*—MRSA (ATCC 43300, ATCC 700699), *Staphylococcus aureus* (ATCC 25923, MTCC 3160), Vancomycin-resistant *enterococcus*-VRE (ATCC 51299) and *Enterococcus faecalis* were used for assessing the antibacterial competence of endophytic fungus. Pathogens were freshly prepared in Tryptic soy broth (TSB) and Brain heart infusion (BHI) broth, respectively. With 0.5 Mc Farland, they were standardized 10^7^–10^8^ colonies forming unit (CFU)/mL.

#### 2.5.2 Agar plug diffusion technique

The agar plug diffusion method (primary antibacterial screening) was conducted for all the fungal endophytes isolated. A sterile cotton swab was used to swab the test microorganisms on the Muller Hinton agar (M173 HiMedia) medium. Using a sterile cork borer, mycelium agar discs (5 mm diameter) were collected from seven days of actively developing pure fungal cultures on PDA media. The plugs were put on MHA plates seeded with the test microorganisms and refrigerated overnight at 4°C to allow metabolite diffusion (in triplicates). The plates were then placed in an incubator for 12 h at 37°C to allow microbial development, and growth inhibition was observed (Marcellano et al., 2017).

### 2.6 Effect of crude extracts on growth of resistant pathogens

#### 2.6.1 Fungal extracts preparation

The 7 days endophytic fungal isolate was cultured in a narrow mouth Erlenmeyer Conical Flask (500 mL Borosil 4,980,024) with Potato dextrose broth- 300 mL (GM403 HiMedia) for 21 days at 27 ± 2°C with regular shaking. The fermented broth and mycelium were separated using filter paper (Whatman No.1). The filtrate was extracted progressively utilizing collective polarity solvents (Hexane, Dichloromethane, Ethyl acetate, and Butanol) using a glass separating funnel (Borosil 6,400). Chemical metabolites were extracted from mycelium and vaporized using a rotatory evaporator (Type: RE100-Pro) at decreased pressure (40–45°C) and kept at −20°C (Palanichamy et al., 2018).

#### 2.6.2 Antibacterial bioassay by agar well diffusion method

The dried-out crude was reconstituted with DMSO for antibacterial testing. On Muller Hinton agar (MHA) plates, the bacterial lawn culture was evenly dispersed with a sterile cotton swab. Wells of about 2 mm in diameter have been created and filled with extracts of various solvents. The well diffusion technique was used to study dilutions of fungal extract ranging from 25 to 100 μg/mL against the test pathogens. The system was incubated at 37°C for 24 h. The impact of fungal extracts on pathogen growth was measured after incubation (mm). The trial was carried out in triplicate. Oxacillin (1 mcg) and Vancomycin (30 mcg) commercial antibiotic discs were used as positive controls, while Dimethyl sulfoxide (5%) was used as a negative control. For large-scale fermentation, fungal isolates with promising antibiotic action were chosen (Anitha and Mythili, 2017).

### 2.7 Morphology and microscopic analysis

The fungal isolate was initially identified using scanning electron microscopy (EVO/18 Research, Carl Zeiss) to examine morphological parameters, spore structure, and surface morphology. The culture development profile, spore colors, and morphologies were examined using standard guides. The fungal strain was characterized by the Lacto phenol cotton blue method and detected in the microscope.

### 2.8 Molecular genomic recognition of endophytic fungus

The species-level identification of the potential isolate was accomplished by utilizing 18S rRNA ITS sequencing. Fungal isolate genomic DNA was attained by NucleoSpin^®^ Tissue Kit. Using ITS1 (5’TCCGTAGGTGAACCTGCGG3’) and ITS4 (5’ TCCTCCGCTTATTGATATGC 3′) universal primers, the 18S rRNA gene was amplified. Initially, denaturation was carried out for 2 min at 95°C, followed by 25 cycles of 30 s of denaturation at 95°C, primer annealing at 55°C for 30 s, first extension for 1 min at 72°C and last extension for 5 min at 72°C were the PCR thermal cycling conditions. The sequencing outcomes were achieved using ABI PRISM^®^ BigDyeTM Terminator Cycle Sequencing Kits with AmpliTaq^®^ DNA polymerase (Applied Biosystems). Using the Blast tool, the attained sequence of isolate PTS8 was matched through the NCBI database. By the neighbor-joining tree technique, the phylogenetic tree was built. The consensus sequences were deposited in GenBank (Gautam et al., 2016).

### 2.9 Minimum inhibitory and minimum bactericidal concentration

The NCCLS-recommended broth microdilution susceptibility assay was used to determine the Minimum Inhibitory Concentration (MIC) and Minimum Bactericidal concentration (MBC) of crude with the greatest inhibitory effect. The entire experiment was conducted in Muller Hinton broth (MHB). To dissolve the fungal extracts, DMSO (10%) was employed, and a 96-well plate dilution was generated from (50 μg/mL–0.39 μg/mL) concentration. Following this, each well received 100 μL of Muller Hinton broth (M391 HiMedia) and 10 μL of preprepared bacterial suspension. The plates were incubated for 24 h at 37°C. The MIC was later determined to be the well with the lowest concentration and no observable growth of microbes. The drugs Oxacillin and Vancomycin were utilized as positive controls for MRSA and VRE, respectively, whereas MHB was used as a negative control. The MIC findings identified the dilutions that had no obvious bacterial growth. 50 μL of broth was transferred from each of these wells to the MHA medium and incubated according to the preceding limitations. MBC was the final concentration that inhibited total bacterial growth. The experiment was performed in triplicates (dos Santos et al., 2015).

### 2.10 MIC index of fungal extract

To confirm the efficacy of ethyl acetate extract whether it is bactericidal or bacteriostatic. By dividing MBC by MIC value, the MIC index was assessed. The MIC index was calculated by dividing MBC by the MIC value. If the index value is less than or equal to 4, it is called bactericidal, and if it is larger than 4, it is considered bacteriostatic (Sadrati et al., 2020).

### 2.11 Synergistic study of crude extract

The checkerboard experiment was used to appraise the fungal extract’s synergistic interaction with antibiotics such as Oxacillin and Vancomycin. Synergistic combinations of the extract and antibiotics were created at the MIC value to which the bacterial strains were resistant, and then serially diluted in two steps. The FICI (Fractional inhibitory concentration index) was calculated using the method below to determine the optimal interaction combination.

$$\begin{array}{l} \mathrm{Fractional inhibitory concentration}\;\left(FIC\right)\;\mathrm{of fungal crude extract}=\\ \mathrm{Extract}\;\mathrm{MIC}\;\mathrm{in combination with antibiotic}/\mathrm{Extract}\;\mathrm{MIC}\;\mathrm{alone}\text{.} \end{array}$$

$$\begin{array}{l} \mathrm{Fractional inhibitory concentration}\;\left(FIC\right)\;\mathrm{of antibiotic}=\mathrm{Antibiotic}\;\\ \mathrm{MIC}\;\mathrm{in combination with extract}/\mathrm{Antibiotics}\;\mathrm{MIC}\;\mathrm{alone}\text{.} \end{array}$$

FICI=AntibioticFIC+FungalcrudeextractFIC.

The effects were inferred as FICI (≤0.5) synergic effects, FICI >0.5 to 4 indicates indifference, FICI >4 represents antagonism (Omokhua et al., 2019).

### 2.12 Qualitative phytochemical screening

The screening was performed for ethyl acetate solvent crude extracts of PTS8 isolate as per standard procedures described by Devi et al. (2012). This method aims to check the presence or absence of Flavonoids, Tannins, Alkaloids, Saponins, Steroids, Cardiac glycosides, and Phenolic compounds in the fungal crude.

#### 2.12.1 Quantitative phytochemical screening

Based on the qualitative examination, a quantitative investigation was carried out for Tannins, Flavonoids, Phenols, and saponins by following (Gokilavani and Banu, 2021).

### 2.13 Gas chromatography–mass spectrometry sample preparation

The obtained active endophytic fungal ethyl acetate dry extract was liquefied with the equivalent solvent and dissolved thoroughly to make a concentration of 1 mg/mL and subjected to GC–MS analysis.

#### 2.13.1 GC–MS analysis of PTS8 fungal crude extract

GC–MS analysis was carried out in Perkin Elmer Clarus-680 equipped with a Clarus 600 mass spectrometer and a capillary column (30 m, 0.25 mm ID, 250 m df). The temperature in the early oven was kept at 60°C for 2 min, then ramped to 300°C at 10°C/min for 6 min. Helium was supplied at a constant flow rate of 1 mL/min, and the mass transfer line and source temperature were set at 240°C. The entire procedure takes 25 min to complete. Turbo mass software (5.4.2 version) was used for the spectrum analysis. The configurations of the chemical were compared to the mass spectral outlines in the NIST collection (2008) (Gautam et al., 2016).

### 2.14 DPPH radical scavenger assay

The anti-oxidative property of the fungal crude extracts was determined *in vitro* using DPPH (2,2-Diphenyl-1-Picrylhydrazyl). For the investigation, fungal extracts were dissolved in DMSO. The extract and standard (Ascorbic acid) were diluted to 50, 100, and 150 μg/mL in a test tube, and 2 mL of DPPH was supplied. The tubes were incubated for 30 min at room temperature in the dark, and absorbance was recorded using a UV–Vis spectrophotometer at 517 nm. The fraction of inhibitory free radicals was calculated using (Soni et al., 2021).

$$\begin{array}{l} \mathrm{Inhibition percentage of free radicals}=\\ \left[\mathrm{Abs}\;\mathrm{of control}-\mathrm{Abs}\;\mathrm{of sample}/\mathrm{Abs}\;\mathrm{of control}\right]\times 100. \end{array}$$

#### 2.14.1 Reducing power assay

The radical scavenging activity of fungal extracts was determined using the reducing power method. Various extract concentrations (50,100,150 μg/mL) were mixed with 2.5 mL of 0.2 M phosphate buffer (pH 6.6) and 2.5 mL of potassium ferricyanide (1%). At 50°C, the mixtures were incubated for 20 min and 2.5 mL of 10% trichloroacetic acid was added. Then it is centrifuged for 10 min at rpm of 1,000. Post centrifugation, the supernatant 2.5 mL was added to 2.5 mL d.H_2_O and 0.5 mL ferric chloride (0.1%) and vortexed. The absorbance was read at 700 nm. Ascorbic acid is used as the reference drug. The test was done in triplicates (Zeng et al., 2011).

$$\mathrm{Percentage}\;\mathrm{inhibition}=\left[A_{\mathrm{control}}-A_{\mathrm{sample}}/A_{\mathrm{control}}\times 100\right]\text{.}$$

Where ‘A’ is the absorbance.

### 2.15 Statistical analysis

Each of the *in vitro* experiments was run in triplicates and the outcomes were estimated by Version 9.5.1 (733) GraphPad Prism software. The data were provided concerning average ± standard deviation. The results were analyzed with one-way ANOVA.

## 3 Results

### 3.1 Isolation and screening of potential endophytic fungi

**Figure 1:**
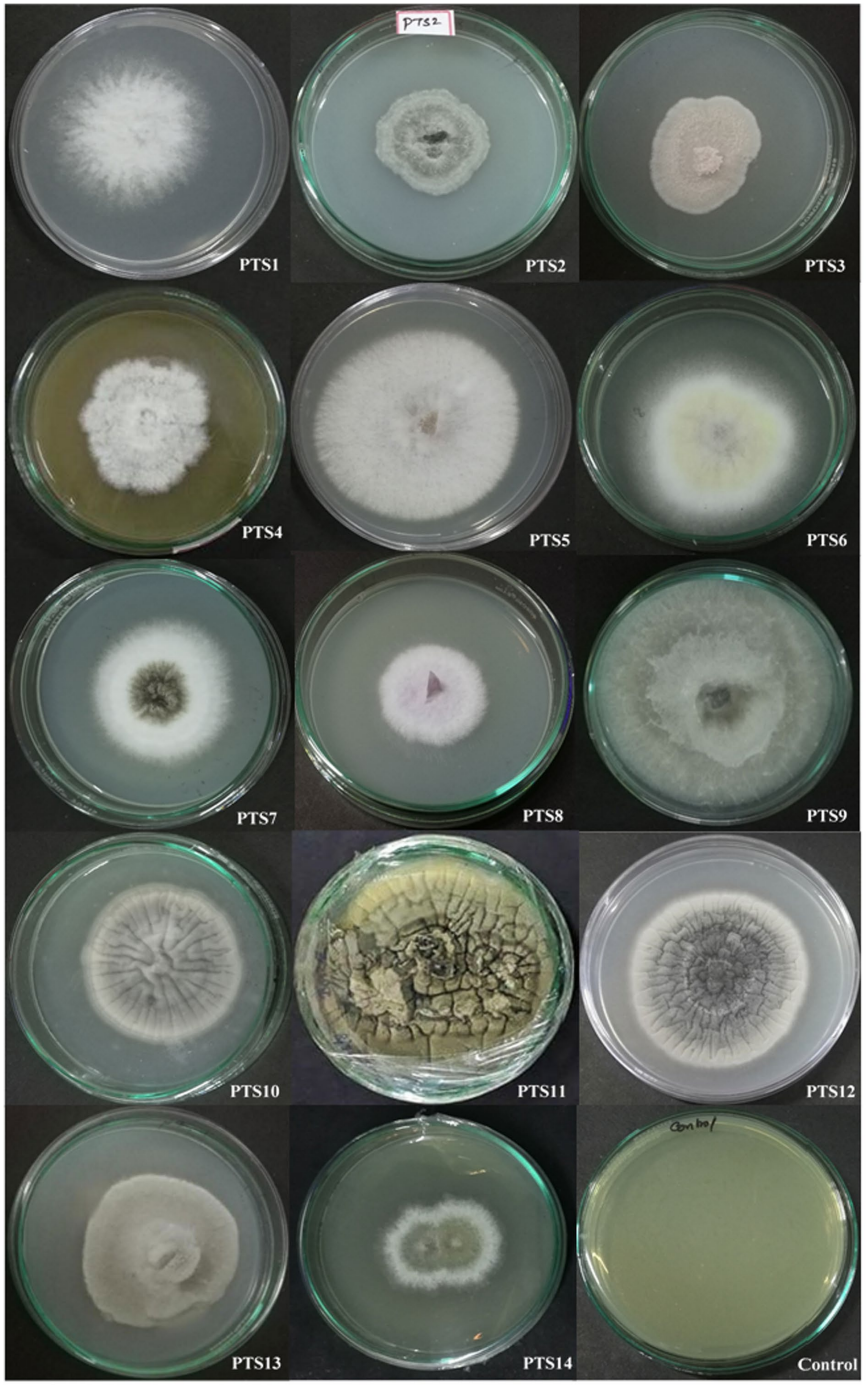
Endophytic fungal isolates derived from the stem of *P. tuberosa*.

**Figure 2:**
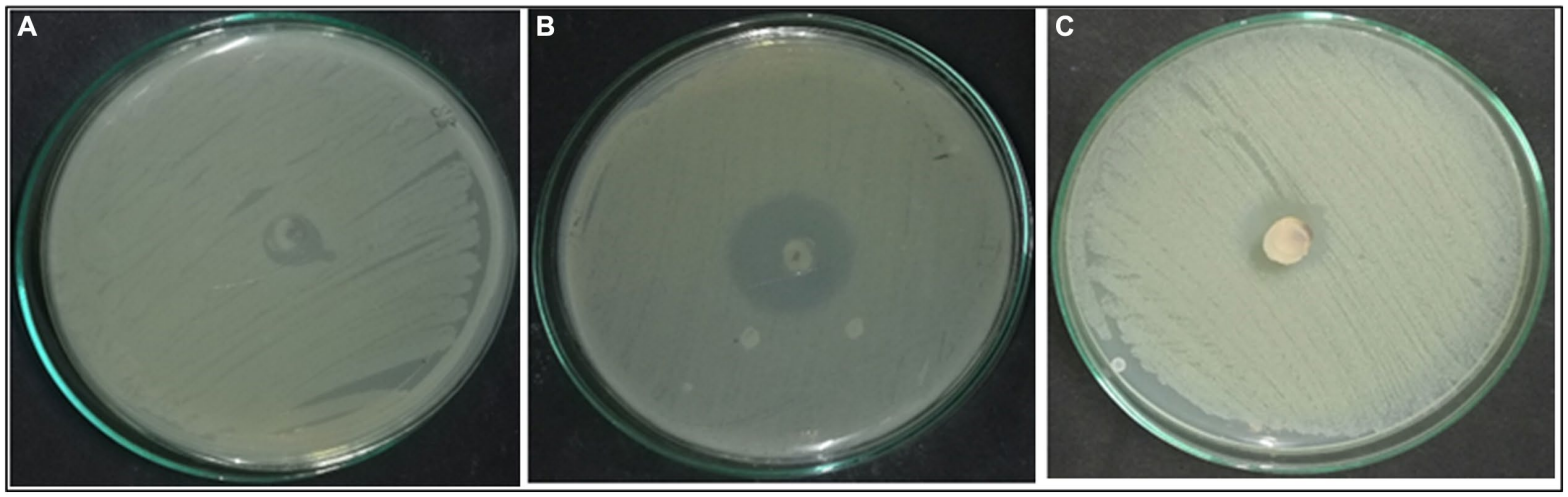
Effect of *Xenomyrothecium tongaense*. Agar plug diffusion—**(A)** MRSA (ATCC 43300), **(B)** ATCC 700699, **(C)** VRE (ATCC 56299).

**Figure 3:**
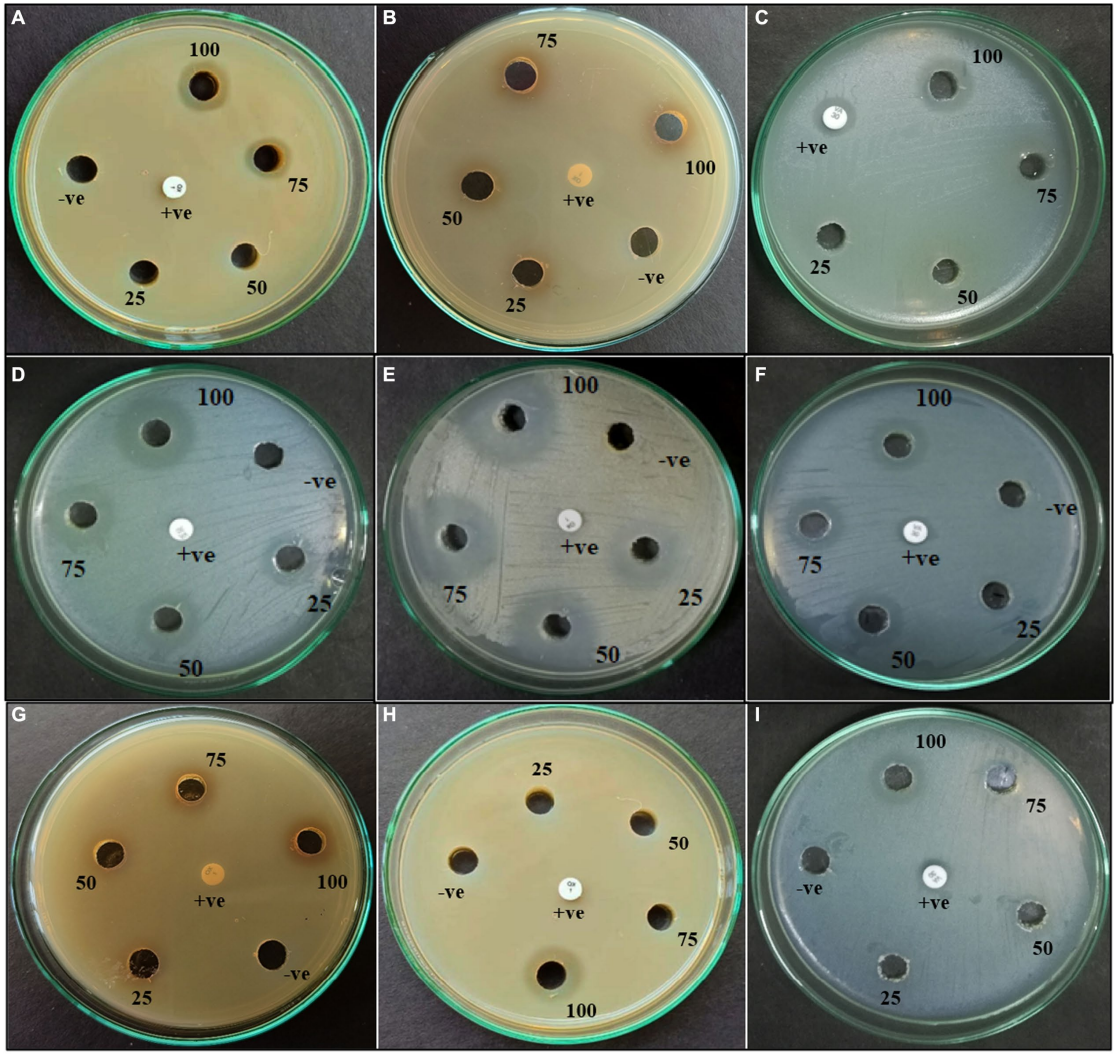
Agar well diffusion of ethyl acetate extract of PTS3—**(A–C)**; PTS8—**(D–F)**; PTS11—**(G–I)** against **(A, D, G)** MRSA (ATCC 43300); **(B, E, H)** MRSA ATCC 700699; **(C, F, I)** VRE (ATCC 56299).

According to morphological characteristics, 14 isolates of endophytic fungi from *P. tuberosa* were obtained from the stem (Figure 1) with no bacterial or fungal growth in the control plate. According to their tissue of origin, the isolates obtained were coded as PTS1-PTS14 from stems. All 14 fungal endophytes were exposed to determine their antibacterial efficacy by the agar plug diffusion technique (primary screening). Three fungal endophytes that showed positive antibacterial activity in initial screening (Table 1) were cultivated independently for 21 days at 27 ± 2°C in potato dextrose broth and extracted with various polarity solvents. Each resulting extract was individually tested for antagonistic ability using the agar well diffusion technique (secondary screening). PTS8 isolate crude extracts were the most active against all of the pathogenic microorganisms tested. The highest antagonism was seen in ethyl acetate extract with inhibitory zones of 20 ± 0.25 mm and 24 ± 0.14 mm against MRSA ATCC 43300 and ATCC 700699, respectively, which was 3 to 4 times greater than the positive control. Inhibition was seen against *S.aureus* ATCC 25923 and MTCC 3160 at 20 ± 0.27 mm and 22 ± 0.47 mm, respectively. At 100 μg/mL concentration, the extract inhibited VRE with an inhibition zone of 18 ± 0.23 mm and *E.faecalis* with an inhibition zone of 21 ± 0.11 mm. The dichloromethane crude had a ZOI of 16–20 mm in diameter against the pathogens tested. Bacterial growth was disrupted when ethyl acetate extract concentrations increased, resulting in bacterial growth inhibition. In contrast, no zone was found for the negative control (DMSO). Table 2 validates the results as the diameter of inhibition. Based on the findings, PTS8 isolate has the potential to be a spectacular antibacterial medication that may be studied further. The results of the ANOVA indicated that there is a significant difference between the means of antibacterial results. The antagonistic activity of the PTS8 isolates in primary and secondary screening is shown in Figures 2, 3.

**Table 1:** Inhibition of pathogens by active isolates isolate of *P. tuberosa*.

Isolates	MRSA (ATCC 43300)	MRSA (ATCC 700699)	*S. aureus* (ATCC 25923)	*S. aureus* (MTCC 3160)	VRE (ATCC 56299)	*E. faecalis*
PTS4	−	+	+	+	−	+
PTS8	+	+	+	+	+	+
PTS13	+	−	+	+	+	+

“+” indicates the presence and “−” absence of a zone of inhibition.

**Table 2:** Antibacterial Susceptibility of the *X. tongaense* secondary metabolites from ethyl acetate crude extract.

Bacterial pathogens	Concentration 100 μg/mL					
	MRSA (ATCC 43300)	MRSA (ATCC 700699)	*S. aureus* (ATCC 25923)	*S. aureus* (MTCC 3160)	VRE (ATCC 56299)	*E. faecalis*
Hexane	16.2 ± 0.05^**^	15 ± 0.1^**^	17.2 ± 0.07^**^	18 ± 0.05^**^	18.5 ± 0.1^**^	20 ± 0.1^**^
DCM	15 ± 0.07^**^	16 ± 0.05^**^	18.5 ± 0.05^**^	18 ± 0.1^*^	12.6 ± 0.05^**^	14 ± 0.05^**^
Ethyl acetate	20 ± 0.25^**^	24 ± 0.14^**^	20 ± 0.27^**^	22 ± 0.47^**^	18 ± 0.23^**^	21 ± 0.11^**^
Butanol	15 ± 0.05^**^	16 ± 0.05^*^	21 ± 0.05^*^	19 ± 0.05^*^	17 ± 0.05^**^	19 ± 0.05^*^
Oxacillin	10 ± 0.4^**^	12 ± 0.2^*^	17 ± 0.1^*^	18.3 ± 0.25^*^	11 ± 0.25^**^	15 ± 0.1^*^
Vancomycin	–	–	–	17 ± 0.05^*^	11 ± 0.03^*^	14 ± 0.05^*^

Values expressed as ZOI (mm) ± SD of Crude extract (*n* = 3). “*” indicates significant differences (*p < 0.05; **p < 0.01) from the control. Positive control-Oxacillin (1 mcg/disc), Vancomycin (30 mcg/disc); Negative control-DMSO (5%); “–” denotes no antibacterial activity.

### 3.2 Identification of potential inhibitory isolate

**Figure 4:**
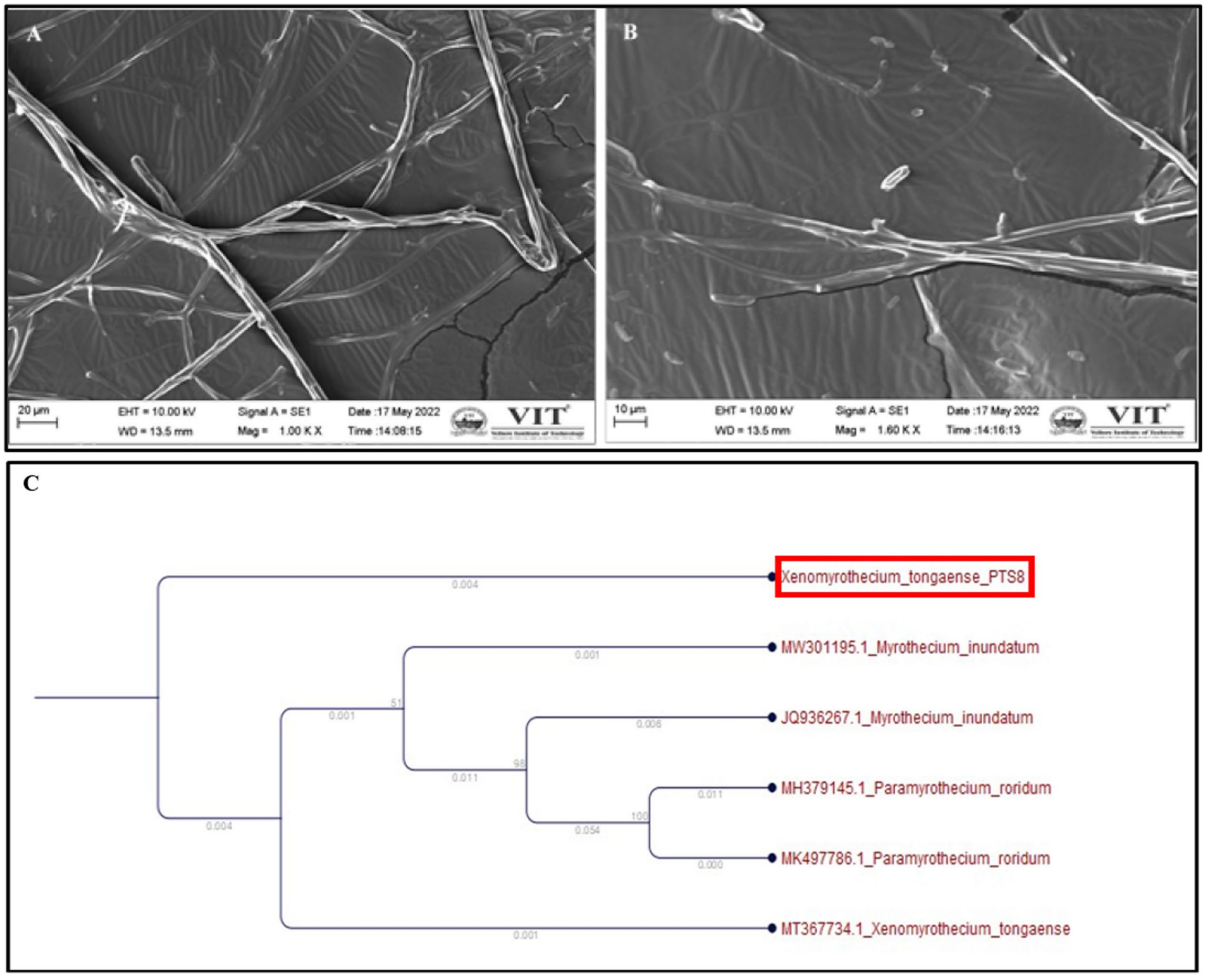
*Xenomyrothecium tongaense* scanning electron micrographs of spores and hyphae **(A)** (scale bar = 20 μm), **(B)** (scale bar = 10 μm). The hyphae were observed at 10,000×, **(C)** Phylogenetic analysis representing *Xenomyrothecium tongaense* PTS8 isolated from the stem of *P. tuberosa* with the neighbor-joining method.

**Figure 5:**
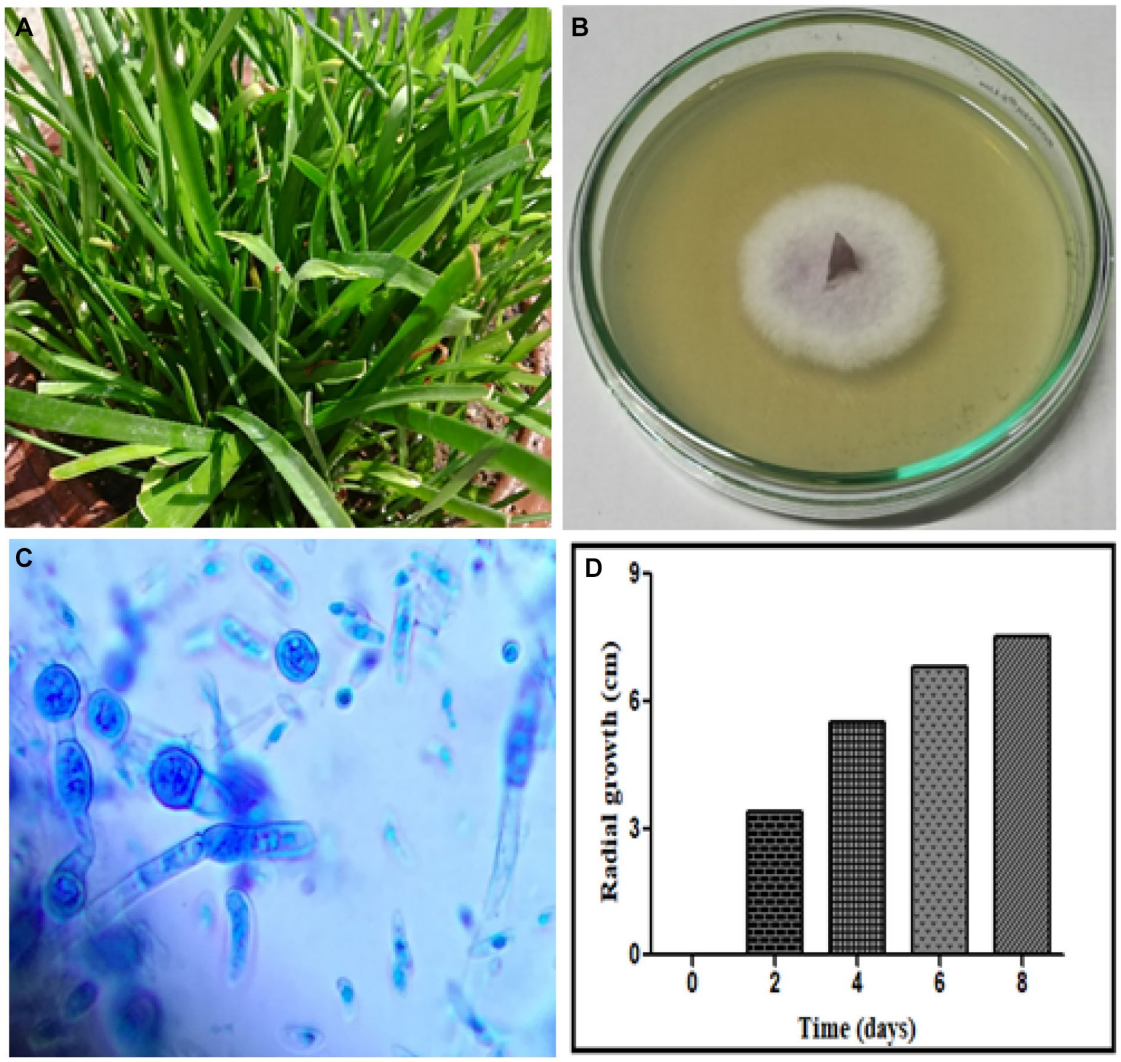
**(A)** *Polianthes tuberosa* plant, **(B)** Mycelial growth of PTS8 isolate from the stem on the PDA medium, **(C)** PTS8 was stained with Lactophenol cotton blue and observed under a light microscope at 100x magnification, Scale bar (0.2 μm), and **(D)** Mycelial growth rate of PTS8.

The fungal isolate PTS8 showed strong antagonistic activity against the tested resistant pathogens and was further characterized morphologically and molecularly. The fungal isolate PTS8 is a fast-growing white fungus that turns purple at the center after 72 h. The potent fungal endophyte PTS8 was identified as *Xenomyrothecium tongaense* by 18 s rRNA ITS sequencing with 98% identity. The resulting sequence was deposited in GenBank with the accession number ON678071. The nucleotide BLAST sequence result was used to construct the phylogenetic tree using the neighbor-joining technique (Figure 4C). Figure 5B depicts the colony form of *Xenomyrothecium tongaense* PTS8 cultivated on PDA. Figures 5C, 4A,B show the microscopic appearance of LCB staining and spore morphology. According to our knowledge, no research has been conducted on the *X. tongaense* endophyte, which inhibits MDR bacterial development.

### 3.3 MIC and MBC of the fungal extracts

The endophytic fungus *X. tongaense* that showed the highest inhibitory effect in the agar well diffusion method was analyzed for its minimum inhibitory and bactericidal effect by a two-fold serial dilution protocol. The MIC values varied from 0.78 to 6.25 μg/mL. The ethyl acetate fungal extract of *X. tongaense* has the highest MIC of 3.12 ± 0.05 and 1.56 ± 0.05 μg/mL against MRSA ATCC 43300 and ATCC 700699, whereas the MIC value was 6.25 ± 0.25 μg/mL against VRE. The antagonistic activity of the extracts seems to be directly proportional to their concentration. Therefore, higher antibacterial substance concentrations yielded a more significant bacterial growth suppression. The MBC of the ethyl acetate extracts from the two dilution units evaluated was above the MIC of the extract. The extract showed a bactericidal effect in the 50–12.5 μg/mL range. The lowest MBC of the extract exhibited complete bacterial growth inhibition was 12.5 ± 0.04 μg/mL for MRSA ATCC 700699 and 25 ± 0.01 μg/mL for VRE. The results of the crude extract against tested pathogens are presented in Table 3.

**Table 3:** MIC and MBC of fungal ethyl acetate crude extract.

Bacterial pathogens	Ethyl acetate crude extract of PTS8		
	MIC (μg/mL)	MBC (μg/mL)	MIC index
MRSA (ATCC 43300)	3.12 ± 0.05*	50 ± 0.01*	16
MRSA (ATCC 700699)	1.56 ± 0.05**	12.5 ± 0.04*	7
*S.aureus* (ATCC 25923)	3.12 ± 0.03*	50 ± 0.2**	16
*S.aureus* (MTCC 3160)	0.78 ± 0.25*	12.5 ± 0.05	16
VRE (ATCC 56299)	6.25 ± 0.25**	25 ± 0.01**	4
*E.faecalis*	3.12 ± 0.05*	25 ± 0.5*	8

MIC index ≤4 is bactericidal and >4 is bacteriostatic, “*” indicates significant differences (*p < 0.05; **p < 0.01) between the treated and the control samples.

### 3.4 Synergistic testing of ethyl acetate extracts

The combination of ethyl acetate fungal crude extract with Oxacillin and Vancomycin was evaluated against test pathogens. Table 4 shows the findings of the chequerboard assay. The MIC value of the fungal extract was 3.12 ± 0.05 and 1.56 ± 0.05 μg/mL against MRSA ATCC 43300, and ATCC 700699, respectively. Whereas, the combination of fungal extract and Oxacillin performed synergistically,drastically lowering the MIC value to1.56 ± 0.05 and 0.19 ± 0.21 μg/mL, respectively. Vancomycin combinations with the extract decreased MIC from 12.5 ± 0.05 to 1.56 ± 0.2 μg/mL with FICI of 0.24 against MRSA (ATCC 43300) indicating a strong synergistic impact (FICI 0.24). Furthermore, the fungal extract showed MIC of 6.25 μg/mL whereas thecombination of fungal extract and Vancomycin acted synergistically against VRE. It decreases the MIC level to 1.56 μg/mL with an FICI of 0.4, demonstrating synergism against VRE (ATCC 56299). Thus, the findings indicate that the fungal extracts exhibit significant synergistic impact when it is combined with the antibiotics than it is used alone may aid in the treatment of infections caused by drug-resistant bacteria.

**Table 4:** Synergistic interaction between fungal crude extract with antibiotics.

Pathogen	Antibiotic	MIC (μg/mL)				FICI	Interpretation
		Extract		Antibiotic			
		Alone	Combination	Alone	Combination		
MRSA (ATCC 43300)	Oxacillin	3.12 ± 0.05*	1.56 ± 0.05*	50 ± 0.25**	0.78 ± 0.04*	0.5	Synergistic
	Vancomycin	3.12 ± 0.05*	0.39 ± 0.11**	12.5 ± 0.05*	1.56 ± 0.2**	0.24	Synergistic
MRSA (ATCC 700699)	Oxacillin	1.56 ± 0.05**	0.19 ± 0.21*	50 ± 0.15**	1.56 ± 0.2**	0.15	Synergistic
	Vancomycin	1.56 ± 0.05**	0.39 ± 0.25*	12.5 ± 0.25**	3.12 ± 0.04*	0.4	Synergistic
VRE	Oxacillin	6.25 ± 0.25**	3.12 ± 0.11**	50 ± 0.5*	12.5 ± 0.03*	0.7	Indifference
	Vancomycin	6.25 ± 0.25**	1.56 ± 0.11**	12.5 ± 0.21**	3.12 ± 0.03*	0.4	Synergistic

FICI ≤ 0.5: synergy, FICI > 0.5 to 4: indifference, FICI > 4: antagonism, “*” indicates significant differences (*p < 0.05; **p < 0.01) between the treated and the control samples.

### 3.5 Mycelium growth rate

The radial mycelial diameter was measured after 7 days of incubation of the colony plates to evaluate the growth rate of the vegetative mycelium. The results showed that the colony’s radial expansion was faster on days 4 and 6 (Figure 5D). According to the study, a nutrient-rich media increases mycelium density and the pace of radial fungal development.

### 3.6 Qualitative secondary metabolite screening

According to the qualitative secondary metabolite analysis, fungal ethyl acetate extracts include phenol, alkaloids, tannins, saponins, and flavonoids. As a result, the existence of these active metabolites serves as a marker that may be used as a pioneer in synthetic drug creation and progress.

#### 3.6.1 Quantitative estimation

Quantitatively the active metabolites present in the ethyl acetate extract of *X. tongaense* PTS8 were screened. The active chemicals estimated quantitatively are represented in Table 5. The results revealed that there is a significant difference between the mean value *p* < 0.05.

**Table 5:** Quantitative phytochemical estimation of the *X. tongaense* PTS8 ethyl acetate extract.

Phytochemicals	Total content (mg/mL)
Phenol	35.20 ± 0.05*
Alkaloids	27.37 ± 0.25*
Tannins	19.63 ± 0.04*
Saponins	10.79 ± 0.11*
Flavonoids	30.66 ± 0.15*

Values expressed as mean ± SD of Crude extract (*n* = 3); “*” indicates significant differences (*p* < 0.05).

### 3.7 Analysis of chemical constituents of the fungal extract by GC–MS

**Figure 6:**
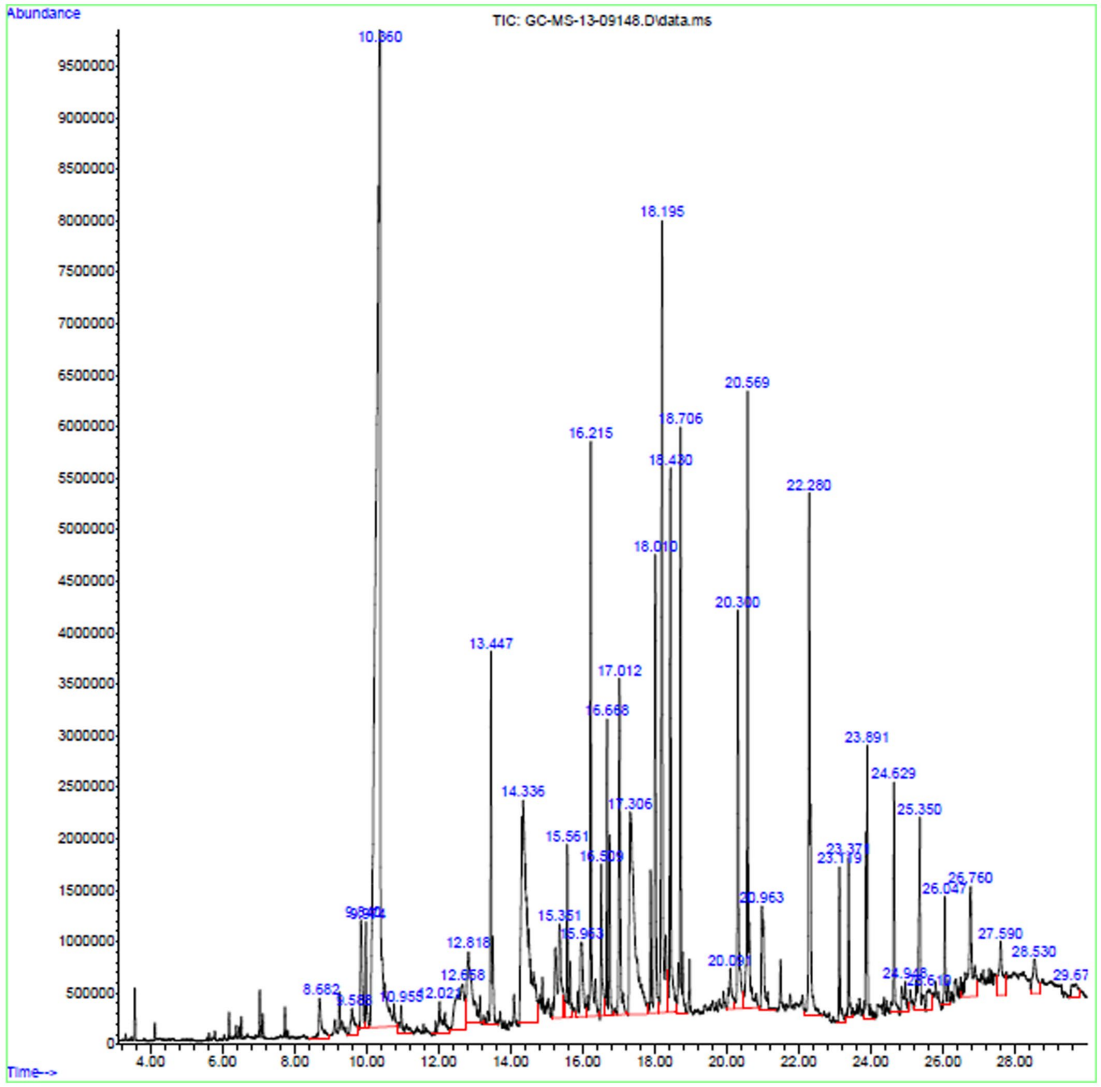
GC–MS chromatogram exploration of ethyl acetate extract of *X. tongaense* PTS8.

The bioactive compounds existing in the extract of *X. tongaense*, which presented the highest inhibitory effect were analyzed by GC–MS. The chromatogram is displayed in Figure 6, and a total of 25 compounds were recognized. Dehydromevalonic lactone (RT 8.6, 0.65%), Benzeneacetic acid (RT 10.3, 22.4%), Pyrrolo[1,2-a]pyrazine-1,4-dione, hexahydro-3-(2-methylpropyl)-(RT 18.19, 6.198%), Octadecanoic acid (RT 20.3, 2.80%), Benzyl Benzoate (RT 16.5, 0.95%), 2-Propenoic acid, tridecyl ester (RT 15.5, 1.22%), Cyclononanone (RT 15.3, 2.07%), n-hexadecanoic acid (RT 18.4, 3.64%), 1-Octadecene (RT 16.6, 2.16%), and 1,2,3-benzenetriol (RT 12.6, 1.81%) were the biochemical substances with antimicrobial activity identified from the selected crude extract. Retention time and area peak revealed a measurable % of estimated biological compounds in the crude.

### 3.8 Antioxidant analysis of fungal extract

**Figure 7:**
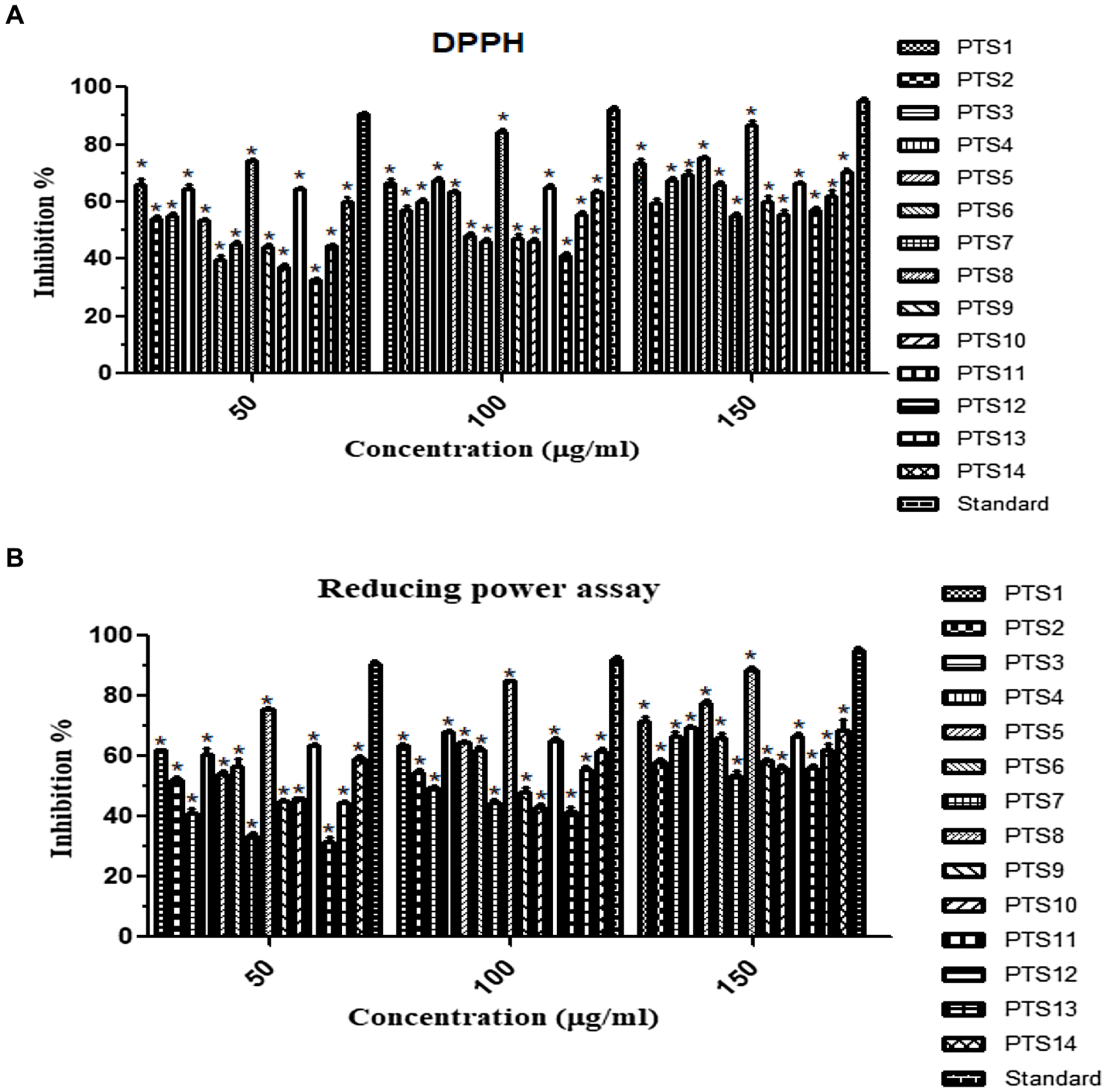
Antioxidant activity of ethyl acetate fungal crude extracts **(A)** DPPH, **(B)** Reducing power assay. “*” represented in the graph indicates *p* < 0.001.

The DPPH and reducing power method was used to determine the antioxidant capacity of all 14 endophytic fungi. The results showed that fungal extracts inhibited free radicals significantly. Furthermore, the percentage of inhibition rise as the crude extract concentration increased (Figure 7). At 150 μg/mL concentration, *X. tongaense* PTS8 ethyl acetate extract has a maximum inhibitory activity of 87 ± 0.5%, whereas ascorbic acid has a maximum inhibitory action of 96 ± 0.5%. The PTS5 isolate had the second-greatest scavenging property, with 75 ± 0.5%. The highest free radical scavenging activity was observed in PTS8 ethyl acetate extract of 88.5 ± 0.5% in reducing power assay. The results of the ANOVA indicated that there is a significant difference between the means of antioxidant results (*p* < 0.001).

## 4 Discussion

The antibacterial activity of the *Xenomyrothecium tongaense* PTS8 isolate isolated by the plug agar technique might be related to the generation of diffusible extracellular metabolites in the agar medium, with these compounds having a specific antibacterial function. This is a straightforward and widely used method for identifying non-volatile compounds produced by bacteria. The fungal endophyte *X. tongaense* PTS8 ethyl acetate extract proved considerably active against the tested MDR human infections. Ethyl acetate was shown to be the most effective organic solvent for extracting fungal bioactive components. According to these findings, the antibacterial bioactive chemicals are semi-polar and may be extracted using ethyl acetate (Sadrati et al., 2020).

Endophytic fungi are taxonomically diverse. These fungi can control the morphological and physiological activity of host plants in a variety of ways. They are found in almost every type of tissue studied and include potential biologically active chemicals. Despite these characteristics, endophytic fungi receive little attention; hence, different ecological functions and biological assets should be extended globally (Du et al., 2022). The ability of endophytic microorganisms, mostly fungi, to create a diverse variety of active compounds that might protect the plant against disease is one of their most important characteristics (dos Santos et al., 2015).

Endophytic fungus *Chaetomium* sp. HQ-1 isolated from *A. chinensis* showed antibacterial action against MRSA with a ZOI of 18 mm in an ethyl acetate extract. 2,6-dichloro-4-propylphenol, differanisole A, and 4,5-dimethylresorcinol pure antibiotic isolate from the fungus was found to display a moderate ability to fight against *L.monocytogenes*, *S. aureus*, and methicillin-resistant *S. aureus* pathogen, with MIC varied 16 to 128 μg/mL (Liu et al., 2019). *Aspergillus niger* derived from *O.ficus-indica* peels displayed a broad-spectrum antibacterial efficacy. The fungus ethyl acetate extract showed prominent action by inhibiting Carbapenem-resistant *Klebsiella pneumoniae* (ATCC BAA-2342) with MIC of 250 μg/mL followed by MIC of 500 μg/mL for both VRE (ATCC BAA-2365) and MRSA (ATCC-700788). MDR *Pseudomonas aeruginosa* (ATCC -BAA-2111) revealed the lowest MIC >1,000 μg/mL (Elkady et al., 2022). *Phomopsis* sp. and *Botryosphaeria* sp., isolated from the leaves and short branches of *Garcinia mangostana* and *Garcinia nigrolineata*, subjected to agar diffusion assay demonstrated significant antagonism against antibiotic-resistant clinical MRSA isolates and *S.aureus* (ATCC 25923), with MIC vary between 32 to 512 μg/mL (Phongpaichit et al., 2006). *S. aureus* and *E.faecalis* were significantly suppressed by *N. sphaerica* (URM-6060) and *Pestalotiopsis maculans* (URM-6061) endophytic fungus of *Indigofera suffruticosa* with an inhibitory zone of 36 and 34 mm (dos Santos et al., 2015). At 1 mg/mL ethyl acetate fungal crude extract concentration, the endophytic fungi *Chaetomium globosum*, *Fusarium* sp. 3, and *Cladosporium ramotenellum* isolated from the plant *Securinega suffruticosa* were shown to have an antagonistic action against *S. aureus* and *E.faecalis* (Du et al., 2022). Aurisin A, a dimeric sesquiterpenoid compound derived from *Neonothopanus nambi* DSM 24013 a wild bioluminescent basidiomycetes exhibited effective antagonism against MRSA ATCC 33591, 43,300, 33,591, and BD 15358, 16,876 clinical strains with MIC of 7.81 μg/mL (Krishnasamy et al., 2023). *Neofusicoccum austral* an endophyte produces novel unsymmetrical naphthoquinone dimer neofusnaphthoquinone B exhibited potent action against MRSA (Cadelis et al., 2021).

A report results, the fungus *Fusarium equiseti*, *Aspergillus niger*, and *Colletotrichum glosporides* were found in the *Sonneratia apetala* (Buch. Ham) of the Sundarbans mangrove forest. This fungus methanol and ethyl acetate crude demonstrated the greatest antibacterial effectiveness, inhibiting *S. aureus* (NCTC 12981) with MIC (0.00024 mg/mL) (Nurunnabi et al., 2020). *Cladosporium* sp., *Talaromyces* sp., *Penicillium* sp., *Lophiostoma* sp., *Eutypella* sp., *Chaetomium* sp., *Purpureocillium* sp., *Pestalotiopsis* sp., *Gongronella* sp., *Pseudallescheria* sp., *Fusarium* sp., *Phyllosticta* sp., and *Scedosporium* sp. are the endophytic fungus isolated from *Eucalyptus exserta* showed remarkable antibacterial activity in bioautography by TLC (thin layer chromatography) with their ethyl acetate extract against *S.auerus* (Mao et al., 2021). Ethyl acetate, Hexane, Chloroform and water extract derived from the fungal endophytes *Penicillium* sp. and *Fusarium moniliforme* which is isolated from the leaves of *Colocasia esculenta* has antagonistic action on drug-resistant pathogens like *E.coli*, *P.aeruginosa*, *Acenitobacter baumanii*, *S.aureus*, *S.typhi* and *K.pneumoniae* (Somwanshi and Bodhankar, 2015). The crude organic extracts obtained from the mycelia of *Xylaria* sp. and *Diaporthe endophytica* from leaves and stems of *Otoba gracilipes* were reported to have inhibitory activity against *E. coli* (ATCC 25922) and *S. aureus* (ATCC 25923) (Charria-Girón et al., 2021).

According to research, mesothelium-like species can generate a bioactive metabolite cocktail with potent antifungal and antibacterial action. More than 50 metabolites from *Albifimbria verrucaria* and *Paramyrothecium roridum* have been identified (Levy and Marshall, 2004). Furthermore, the fungus *Myrothecium* sp. OUCMDZ-2784 produced from the salt-resistant Apocynaceae family Apocynaceae growing in the Yellow River Delta has been utilized to treat heart failure and hypertension. Sesquiterpenes, cyclopeptides, trichothecenes, and diterpenes produced from *Myrothecium* sp. were also shown to exhibit antibacterial and cytotoxic properties. At a concentration of 286 μg/mL, an ethyl acetate extract derived from *Myrothecium* sp. OUCMDZ-2784 suppressed α-glucosidase by 75% (Xu et al., 2018). Alkylresorcinol, myrothecol A (1–3) substances isolated from ethanolic extract of *Myrothecium* sp. GY170016 has also been described. Mycelial mat was shown to be cytotoxic to the MCF-7 human cell line, with IC50 values of 16.7, 13.2, and 21.3 μm (Janeš et al., 2007). The EtOAc crude derived from *Xenomyrothecium* sp. isolate from marine sponge yielded compounds such as TMC-256A1, (3S)-6-hydroxy-8-methoxy-3,5-dimethyl-isochroman, trans-3,4-dihydro-3,4,8-trihydroxynaphtalen-1(2H)-one, 8-hydroxy-6-methyl-9-oxo-9H-xanthene-1-carboxylate, (3R,4S)-4-hydroxymellein, ɷ-hydroxyemodin, and (3R,4R)-4-hydroxymellein (Quang et al., 2020).

A study reported that GC–MS analysis of *Shepherdia argentea* (Pursh.) Nutt. revealed the presence of Pentacosane, which has antibacterial action (Konovalova et al., 2013). Likewise, a 1,2,3-benzenetriol molecule produced from *Quercus cortex* (Oak bark) extracts showed considerable antibacterial activity against *Chromobacterium violaceum* (Deryabin and Tolmacheva, 2015). Due to the synergistic effect of two phenolic metabolites 2,4-Di-tert-butyl-phenol and p-tert-butylcalix[4]arene, MDR bacteria *Pseudomonas aeruginosa* (NR_0754828.1) and *Staphylococcus aureus* (NR_075000.1) were susceptible to *Bacillus licheniformis* isolated from Algerian Hot Spring (Aissaoui et al., 2019). It has been observed that dehydromevalonic lactone produced from Monascus ruber during the fermentation process in a solid state imparts aroma (Anisha and Radhakrishnan, 2017). *Daldinia eschscholtzii*, an endophytic fungus, produced compounds 2,4-Di-Tert-Butylphenol that lower virulence and quorum sensing in *Pseudomonas aeruginosa*. The bactericidal experiment with 2,4-DBP and ampicillin demonstrated synergistic action against *P. aeruginosa*. Furthermore, 2,4-DBP inhibited *P. aeruginosa* adhesion and invasion in A549 lung alveolar cancer cells (Mishra et al., 2020). *Centaurea* essential oils are widely recognized for their medicinal effects. Essential oils including phytol, heptacosane, nonacosane and pentacosane exhibited a beneficial association with antibacterial activity, with excellent antimicrobial action against *S. aureus* and *S. epidermidis*, *E. coli*, *B. cereus* and *K. pneumonia* (Carev et al., 2023). *Streptomyces* sp. KCA1 isolated from leaves of *Phyllanthus niruri* yielded the bioactive compound 2,4-Di-tert-butylphenol (2,4-DTBP). The active compound exhibited antagonistic activity against *S. aureus* ATCC 29213 and *E. coli* ATCC 25922 at MIC values of 0.78 μg/mL and 50 μg/mL. Against the normal VERO cell line and breast cancer cell line (MCF7), the IC50 value of 2,4-DTBP was found to be 116.8 μg/mL and 11.0 μg/mL, respectively, (Seenivasan et al., 2022). The phytocompounds Octadecanoic acid, Hexadecanoic acid, and 2,3-dihydroxypropyl ester were commonly present in the ethyl acetate extracts obtained from *Chaetomium globosum*, *Cladosporium tenuissimum* and *Penicillium janthinellum* endophytic fungus (Kanjana et al., 2019).

Metabolites obtained from *Zingiber officinale* rhizome endophytic fungus GFV1 fungal sp. (KX247125) isolated from layanikkara variety exhibit antibacterial activity against *S. aureus* (MTCC 96), *B. subtilis* (MTCC 121) and *Salmonella enterica Typhimurium* (MTCC 1251) with inhibition of 20, 10, and 10 mm, respectively. It also demonstrated antifungal activity against *Pythium myriotylum* on dual culture method with inhibition of 53.3%. Their fungal extracts also produced bioactive compounds such as benzene acetic acid, dehydromevalonic lactone and n-hexadecanoic acid which was identified by GC–MS. The fungal endophyte *Chaetomium fusiforme* also reported the presence of a Benzene acetic acid molecule (Anisha and Radhakrishnan, 2017). A study reported that purified Benzyl Benzoate compound from the toluene fraction obtained from *Emericiella quadrilineata*-derived fern *Pteris pellucida* showed significant antagonism against *Aeromonas hydrophilla* and *S. aureus* at a zone of inhibition of 22 ± 0.8 15 ± 0.8 in disc diffusion assay. GC–MS analysis of toluene fraction disclosed the presence of benzyl benzoate (27.3%). Medically benzyl benzoate is an active ingredient of Ascabiol that is generally used as an ointment to treat scabies and various skin diseases (Goutam et al., 2016). Propenoic acid, tridecyl ester, a bioactive molecule produced by *Paecilomyces* sp. (JN227071.1) endophytic fungus reported to exhibit antifungal action against *Rhizoctonia solani* (Hawar et al., 2023). As a result, the current study focuses on the antibacterial properties of *Xenomyrothecium tongaense* to battle multi-drug-resistant bacterial infections.

## 5 Conclusion

Our study reports highly novel findings of (1) endophytic fungi from the plant *P.tuberosa*; (2) the endophytic nature of the fungi *Xenomyrothecium tongaense* PTS8; and (3) the antibacterial efficacy of the isolated endophytic fungus *X. tongaense* PTS8 against MDR pathogens. This is the first report on the endophytic fungus association in the stem of the *P. tuberosa* plant and the first report on the endophytic nature of *Xenomyrothecium tongaense* fungus. Also, this is the first report to study the antibacterial activity of *X. tongaense* to battle multi-drug-resistant bacterial human pathogens. Moreover, the *X. tongaense* fungal extract was significantly inhibiting the MDR pathogens at its minimal extract concentration and it is more potent than the positive control used. The present research will contribute to the application of endophytic fungus as a potent antibacterial agent for enhanced human infectious disease management. Further, the limitations of the study are analytical chemistry investigation on purification and identification of the bioactive secondary metabolite and its mechanistic involvement in suppressing the growth of drug-resistant infections are yet to be done. *In vitro* and *in vivo* toxicity and efficacy of the crude extract are yet to be validated in future studies. In addition to laying the groundwork for future research, this study examines the possibility of producing effective antibacterial drugs to tackle drug-resistant bacterial illnesses.

## Data availability statement

The original contributions presented in the study are included in the article/supplementary materials, further inquiries can be directed to the corresponding author.

## Author contributions

RS: Writing – original draft, Writing – review & editing. SA: Supervision, Writing – review & editing.
